# Insights into Early Onset Colorectal Cancer through Analysis of Normal Colon Organoids of Familial Adenomatous Polyposis Patients

**DOI:** 10.3390/cancers14174138

**Published:** 2022-08-26

**Authors:** Matthew A. Devall, Stephen Eaton, Mourad W. Ali, Steven M. Powell, Li Li, Graham Casey

**Affiliations:** 1 Center for Public Health Genomics, University of Virginia, Charlottesville, VA 22908, USA; 2 Department of Family Medicine, University of Virginia, Charlottesville, VA 22903, USA; 3 Digestive Health Center, University of Virginia, Charlottesville, VA 22903, USA; 4 Comprehensive Cancer Center, University of Virginia, Charlottesville, VA 22911, USA; 5 Department of Public Health Sciences, University of Virginia, Charlottesville, VA 22908, USA

**Keywords:** familial adenomatous polyposis, early onset colorectal cancer, colon organoid, RNA-seq

## Abstract

**Simple Summary:**

The increase in early onset colorectal cancer (EOCRC) rates places a new, heavy burden on a younger population. Current studies in EOCRC are often affected by factors such as increased cellular heterogeneity, which mask biological signal. We overcome this using the colon organoid model, which is composed of cells from which CRC is believed to originate. We perform gene expression analysis on colon organoids derived from healthy and familial adenomatous polyposis patients, who are genetically predisposed to develop CRC at a young age, to identify differences that may be used for early monitoring of CRC development. We contextualize our findings in the framework of existing CRC and EOCRC data. We also show that ethanol, a CRC risk factor, may play an important role in further driving aberrant gene expression at EOCRC relevant genes. Functional analysis of these genes may shed new insight into EOCRC.

**Abstract:**

Early onset colorectal cancer (EOCRC) rates have increased in recent decades. While lowering the recommended age for routine colonoscopies to 45 may reduce this burden, such measures do not address those who develop CRC before that age. Additional measures are needed to identify individuals at-risk for CRC. To better define transcriptomic events that precede the development of CRC, we performed RNA-sequencing analysis in colon organoids derived from seven healthy and six familial adenomatous polyposis (FAP) patients. This led to the identification of 2635 significant differentially expressed genes (FDR < 0.05). Through secondary analysis of publicly available datasets, we found that these genes were enriched for significant genes also present in FAP CRC and non-hereditary CRC datasets, including a subset that were unique to EOCRC. By exposing FAP colon organoids to a three-day ethanol treatment, we found that two EOCRC-relevant genes were also targets of CRC related lifestyle factors. Our data provides unique insight into the potential, early mechanisms of CRC development in colon epithelial cells, which may provide biomarkers for patient monitoring. We also show how modifiable lifestyle factors may further alter genes relevant to EOCRC, adding weight to the hypothesis that such factors represent an important contributor to increased EOCRC incidence.

## 1. Introduction

Colorectal cancer (CRC) is a complex, heterogenous disease with a strong genetic component [1,2,3]. Overall, the incidence of CRC has decreased steadily since the mid 2000’s. A decline that has largely been attributed to improved adherence to CRC screening programs [4]. In contrast, there has been an alarming increase in the incidence of early onset CRC (EOCRC) (defined as <50 years old) [4,5]. The initial increase in EOCRC in the US was primarily driven by rectal cancer, but since 2012, incidence of cancers of both colon and rectum have been increasing at similar rates [6]. This increase in EOCRC places a significant burden on a younger population, where screening has not been routinely recommended until recently [7]. While it is now recommended that individuals begin by the age of 45, this does little to aid those who develop CRC at a younger age, where increased incidence has also been reported [8]. 

The vast majority of EOCRCs are believed to be driven by non-hereditary forms of CRC [9]. While the causes of these ‘sporadic’ EOCRC remain poorly understood, generational shifts in environment, lifestyle, and dietary habits have been strongly implicated [10]. For example, a recent study has provided evidence that moderate to high consumption of alcohol is associated with a greater risk of EOCRC than late onset CRC [11]. However, approximately one fifth of patients with EOCRC are affected by a hereditary cancer syndrome [4], which is about four times greater than the rates of these syndromes in CRC across all ages. Familial adenomatous polyposis (FAP) is a hereditary syndrome that leads to colon cancer. It is driven by inherited or de novo mutations in the tumor suppressor gene APC regulator of WNT signaling pathway (*APC*) [12]. Patients with FAP typically develop hundreds of adenomatous polyps in their late teens or early twenties [13]. If left untreated, the development of CRC is almost inevitable and develops at an average age of 39 [12,14]. While somewhat rare in the general population (1/7500–1/10,000) [13], FAP is the second most common, hereditary CRC syndrome [15], accounting for approximately one percent of all CRC cases [12]. Discovery of early aberrant gene expression patterns in uninvolved colons of FAP patients has the potential to provide critical insight to improve our understanding of early events of EOCRC oncogenesis. 

A common limitation in EOCRC research is that the majority of studies have been performed in tumors; for example, within the context of identifying gene expression differences between tumor versus normal-adjacent tissue (NAT) [16]. While this has improved our overall understanding of molecular changes in EOCRC and CRC tumors as a whole, tumor and cellular heterogeneity, increased somatic mutation burden and gross alterations in copy number landscapes in CRC tumors present obstacles for the interpretation of gene expression differences that drive EOCRC. While the incident of EOCRC is rising, EOCRC remains a relatively rare disease and data from few studies on EOCRC tumors have made publicly available, limiting further advancement in the field. Importantly, there is also a lack of well-defined models for the study of EOCRC. Given these limitations, we sought to make use of the colon organoid system (which present with reduced cellular and tumoral heterogeneity) derived from “normal appearing” colon biopsies of FAP patients (i.e., biopsies taken from sites adjacent to polyps during surveillance colonoscopies from patients without known, existing CRC but with extensive polyposis) in an attempt to improve understanding of early events of EOCRC and CRC as a whole. 

Colon organoids represent a model of the colon stem cell niche, where CRC is believed to originate [17]. Previously, our group has used this model to investigate how differences in CRC environmental risk factors may alter gene expression of genes implicated in CRC [18,19,20,21,22]. In this study, we have applied the colon organoid model to identify gene expression differences between FAP subjects and healthy controls. Given the limited available models for EOCRC, we hypothesize that the FAP colon organoid model may be used to gain unique insight into EOCRC and CRC development. Specifically, we aimed to identify CRC-related events in at-risk subjects without existing CRC. Identification of these early biological markers could lead to improved early detection of CRC, including in younger population.

## 2. Materials and Methods

### 2.1. Patient Selection and Biopsy Collection

Healthy subjects (*n* = 7) undergoing standard of care colonoscopy were recruited at the University of Virginia alongside FAP (*n* = 6) who were undergoing surveillance colonoscopies. All healthy subjects included in the study presented with 3 or fewer polyps and no personal or immediate family history of CRC. FAP patients were defined by clinical presentation and/or genetic mutation. FAP and healthy colon organoids displayed no statistically significant differences for age, biological sex or smoking status, though two of the seven healthy subjects were current or previous smokers. All subjects were self-reported to be non-Hispanic White.

### 2.2. Organoid Establishment and Passaging

This study included organoids derived from the left colon for both healthy controls and FAP patients. Biopsy samples were first washed 3 times with DPBS at room temperature. Following this, 10 mL of 9 mM EDTA in room temperature DPBS was added to each biopsy. Biopsies were incubated in DPBS/EDTA for 20 min at room temperature. During this period, samples were manually inverted every 2–3 min. To avoid crypt adhesion, all tubes, tips and pipets were conditioned with wash media (DMEM/F-12, 10% FBS, 10 mM HEPES, 2 mM L-glutamine, 1X Pen-Strep (100 U/mL penicillin, 100 μg/mL streptomycin), 1X Glutamax) during the incubation period. Following incubation. DPBS/EDTA was removed. 10 mL of DPBS without EDTA was added. This volume was then mixed 8–10 times by manual pipetting. After the tissue settled, any supernatant was collected in a 15 mL tube. This process was repeated a total of 3 times. The supernatant was spun at 1200 rpm at 4 °C for 5 min. All but 2 mL of supernatant from each 15 mL tube was then removed and combined in a clean 50 mL tube. Care was taken not to disturb the pellet. Using a conditioned 5 mL pipet, the remaining 2 mL from each tube was then combined into one 15 mL tube, which was then spun at 1200 rpm at 4 °C for 5 min. Following this, the supernatant was removed. 10 μL of complex media (45% Wash Media, 50% L-WRN conditioned media, 10 nM Gastrin, 10 μM Y27632, 1X B27 Supplement, 1X N2 Supplement, 1 mM n-Acetylcysteine, 50 ng/μL EGF, 10 mM Nicotinamide, 500 nM A83, 10 μM SB202190) was added. To reduce the clumping of crypts, the cell solution was mixed through manual pipetting. 200 μL of Matrigel was then added and mixed and 35–50 μL of each sample was plated at the center of wells of a 48-well plate. Matrigel containing cells was then incubated at 37 °C for 15 min. Following this, 300–500 μL of complex media was added and samples were returned to the incubator. Organoids were checked and fed after 24 h. Upon establishment, organoids were subsequently passaged as needed (every 3–5 days) and then frozen. A subset of FAP and healthy colon organoids were imaged during routine passaging using Lumenera Infinity2-2C 2.0 Megapixel CCD Color Camera (Lumenera, Ottawa, Canada, cat. #95107) and Infinity Analyze software version 6.5.5 (Lumenera) at 100× magnification.

### 2.3. RNA Harvesting, Library Preparation, Sequencing and Pre-Processing of Colon Organoid RNA-Seq Data

The NucleoSpin RNA Mini kit (Macherey-Nagel, Düren, Germany) was used to extract total RNA and RNA integrity numbers (RIN) were measured using the Agilent 4200 Tapestation (Santa Clara, United States, cat. G2991BA). RIN values of samples considered within this study ranged from 9.3–10. No significant differences in RIN values were observed between the two phenotypes. Libraries preparation was carried out using the Illumina Stranded mRNA Prep kit. Sequencing of RNA libraries was performed in accordance with standard Illumina protocols within the Genomics Core Facility of the Center for Advanced Genomics Technology (Icahn School of Medicine at Mount Sinai, New York). Libraries were pooled and 100 bp, paired-end reads were sequenced on a NovaSeq 6000. FASTQ files were aligned to GENCODE v29 [23] using STAR (v2.6.1d) [24] and RSEM (v1.3.1) [25]. 

### 2.4. Collection of Publicly Available Data

Publicly available data in the form of raw count matrices were downloaded for our secondary analysis. Matched tumor versus NAT data of human FAP patients was downloaded from Gene Expression Omnibus [26], accession number, GSE153385 [27]. Similarly, raw count files from The Cancer Genome Atlas Colon Adenocarcinoma cohort (TCGA-COAD) [16] were downloaded using the R package, TCGAbiolinks [28]. For analysis of TCGA RNA-seq data, subjects were split into two categories: those who developed CRC before the age of 50 (*n* = 5 pairs; 40–48 y/o; herein TCGA-EOCRC) and those who developed CRC at or after the age of 60 (*n* = 34 pairs; 60–90 y/o; herein TCGA-60+). Data on subjects diagnosed between the ages of 50–59 were excluded from our study. 

### 2.5. Statistical Analysis of RNA-Seq

Gene expression analysis was carried out in R (version 4.1.1) [29]. For novel, FAP versus healthy colon organoid data, gene abundance estimates from RSEM [25] were converted to gene counts using tximport [30] and differential expression between groups was tested by fitting negative binomial generalized linear regression models for each gene in DESeq2 [31]. Genes were considered significant if they survived a 5% adjusted false discovery rate (FDR) in DESeq2 [31]. Regression models on phenotype were corrected for biological sex and age. Pathway analysis was performed by uploading significant (FDR < 0.05) DEGs to STRING under default settings [32]. Pathway reporting was limited to Gene Ontology (GO) [33] and Kyoto Encyclopedia of Genes and Genomes (KEGG) [34] enrichment terms. For data visualization, the lfcShrink() function of DESeq2 was performed while specifying apeglm [35]. A volcano plot was then generated using the EnhancedVolcano package on these adjusted results [36]. For matched, tumor versus NAT data, a paired regression model was considered in DESeq2 [31] while using sample pairing as a blocking factor to adjust for inter-individual differences. 

### 2.6. Ethanol Treatment of FAP Colon Organoids

Ethanol exposures of FAP colon organoids (*n* = 6) were conducted as previously described [19,20]. FAP colon organoid lines were grown in 48 well plates at a density of approximately 10^5^ cells per well. Organoids were treated with fresh growth media plus ethyl alcohol (Sigma Aldrich, St. Louis, United States, cat. E7023) (2 μL per 1 mL of growth media). Organoids within the control group were instead treated with fresh media plus 2 μL cell culture grade distilled water as a control. To replace evaporating ethanol, media was replaced every 24 h for three days with fresh ethanol or water control. For consistency, this dose and timeframe were chosen specifically to determine whether the effects of ethanol previously described in normal colon organoids [19,20], could also be identified in colon organoids derived from FAP patients. In previous studies, this dose was chosen to reflect the circulating alcohol levels in blood of alcoholics and was similar to the dose of blood alcohol levels determined through in vivo studies of rats consuming between 2–4 drinks [37,38]. 

### 2.7. Quantitative PCR

For technical validation of selected significant findings in colon organoids, existing RNA was considered. For replication of findings in matched left colon biopsies (*n* = 4), RNA was extracted using an AllPrep RNA kit (Qiagen, Hilden, Germany, cat. 80284), followed by DNAse treatment using an RNA Clean and Concentrator kit (Zymo, Irivine, United States, cat. R1080). The following gene assays were considered for technical validation and replication of findings in colon organoids and biopsies: NKD inhibitor of WNT signaling pathway 1 (*NKD1*; Hs01548773_m1), EYA transcriptional coactivator and phosphatase 2 (*EYA2*; Hs00193347_m1), early growth response 2 (*EGR2*; Hs00166165_m1), *Ependymin related 1 (EPDR1*; Hs01556067_m1) and IGF like family member 1 (*IGFL1*; Hs01651089_g1). A subset of genes were also analyzed in six FAP colon organoids exposed to ethanol (DNA damage regulated autophagy modulator 1 (*DRAM1*; Hs01022842_m1), growth differentiation factor 11 (*GDF11*; Hs00195156_m1), Notum, palmitoleoyl-protein carboxylesterase (*NOTUM*; Hs00394510_m1) and pleckstrin homology domain containing B1 (*PLEKHB1*; Hs00943921_m1)) for a period of three days. For each analysis, cDNA was synthesized from 2 μg of total RNA using the High-Capacity Reverse Transcriptase cDNA kit (Thermo Fisher) and quantitative real time polymerase chain reaction (qPCR) was performed using the TaqMan Gene Expression Master Mix (Thermo Fisher, Waltham, MA, USA, cat. 4444557) on selected TaqMan assays. Hypoxanthine phosphoribosyltransferase 1 (*HPRT1*; Hs02800695_m1) was used as an internal control gene. The PCR reactions were all ran in duplicate, and were subsequently analyzed using QuantStudio 5 (Thermo Fisher). Reactions were normalized using the control gene *HPRT1,* and calculations were performed according to the 2^−ddCT^ method. For the analysis of expression of FAP biopsy samples (*n* = 2), log fold differences were estimated for each gene in each FAP sample, by comparing the resultant values of that gene to that of the average of the resultant from colon biopsies of two healthy controls. For technical replication of findings, data were analyzed for statistical differences using a linear regression model on log normalized values while adjusting for age and gender.

## 3. Results 

### 3.1. Defining the Transcriptomic Landscape of Colon Organoids of FAP Patients versus Those of Healthy Subjects

**Figure 1:**
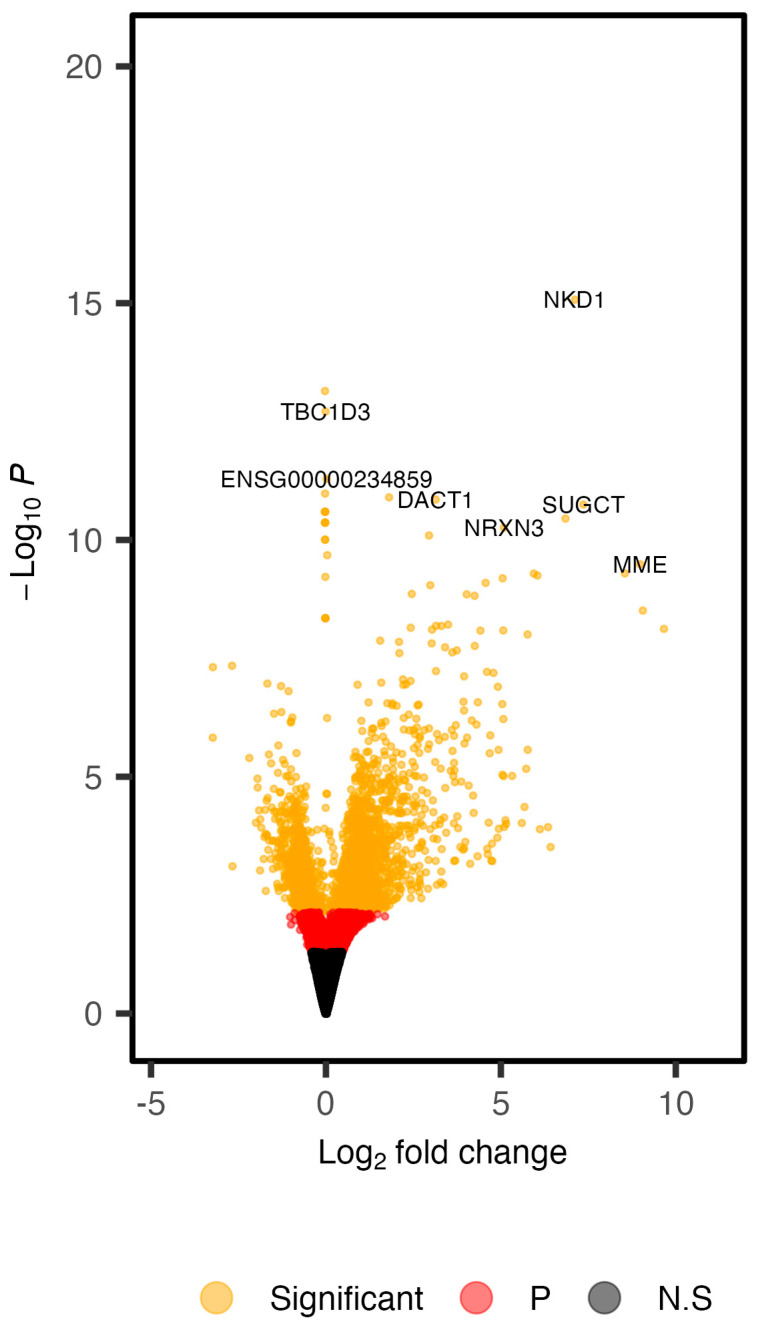
Summary of differential expression findings from our analysis of FAP versus healthy colon organoids. Genes were separated into three categories. Black genes (N.S) were those that did not reach any significance level. Red genes reached nominal significance, but did not survive multiple testing corrections. Generalized linear models were fit for each gene in DESeq2 and a Wald test was used for the identification of significant DEGs. 2635 DEGs (orange) genes remained significant following FDR correction (FDR < 0.05). Positive log2 fold changes represent genes that were increased in FAP versus healthy colon organoids.

**Figure 2:**
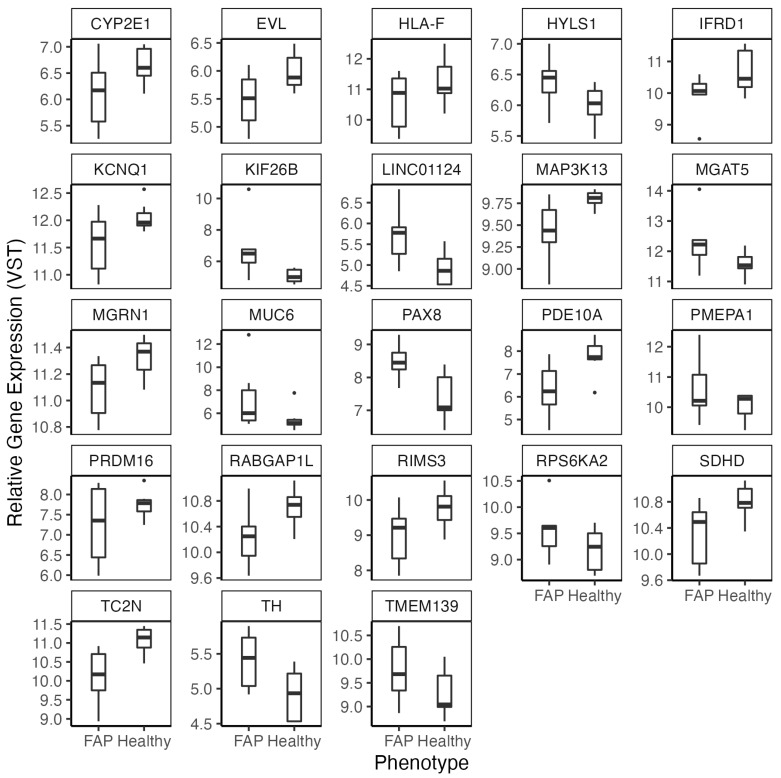
Boxplots of significant DEGs (FDR < 0.05) identified in our analysis (*n* = 13) that were previously found to be associated with the FAP phenotype through DNA methylation analysis of colon organoids. Generalized linear models were fit for each gene in DESeq2 and a Wald test was used for the identification of significant DEGs. To aid in visualization in the plotting genes, relative gene expression was calculated using the VST function in DESeq2.

We compared gene expression of colon organoids generated from FAP patients (*n* = 6) and healthy subjects (*n* = 7). No significant differences for age, sex or smoking status were identified between the two phenotypes (Supplementary Table S1). We generated RNA-seq data on colon organoids from all subjects. An average mapping efficiency of 80.63% was observed, which led to an average of 33.26 million uniquely mapped reads per sample (Supplementary Table S2). Regression analysis was carried out in DESeq2 [31], while accounting for age at the time of colonoscopy and biological sex. This led to the identification of 2635 significant DEGs (FDR < 0.05), 59.66% of which displayed increased expression in colon organoids of FAP versus healthy subjects (Figure 1). Differentially methylated regions previously shown to be unique to EOCRC tumors corresponded to a subset of 23 of these genes (Figure 2) [39].

Pathway enrichment analysis was performed on all DEGs identified in our analysis using the STRING database [32]. Here, we noted that our DEG list was enriched for protein–protein interactions (*p* < 1.0 × 10^−16^), adding weight to their biological relatedness. DEGs were enriched for 104 gene ontology terms [33,34], as well as three KEGG terms: “Wnt signaling pathway” (FDR = 5.30 × 10^−5^), “Hippo signaling pathway (FDR = 3.80 × 10^−3^) and “mitophagy—animal” (FDR = 0.016) (Supplementary Table S3). Extracellular Wnt sensitivity and/or Wnt signaling has previously been suggested to be important for earlier onset of CRC development in other studies [40,41]. 

### 3.2. Relationship of DNA Methylation and Differential Expression in FAP Colon Organoids

Previously, we performed DNA methylation analysis (Illumina Infinium MethylationEPIC Kit) on a small (*n* = 23), largely overlapping (84.62%) cohort of FAP and healthy colon organoids [42]. We identified 358 FDR corrected, differentially methylated regions between FAP and healthy colon organoids, corresponding to 439 unique genes. Of these, 36 genes were found to also be significantly different in our current RNA-seq analysis (Supplementary Table S4), providing further evidence for an important role of these genes in FAP epithelial cells. 

### 3.3. Defining the Overlap in Tumor-Related Gene Expression between FAP and EOCRC

We next defined the transcriptomic landscapes of TCGA-EOCRC and FAP tumors using publicly available data. We performed paired regression analysis within each age grouping to identify DEGs between tumor versus NAT for each of the two subsets. A remarkable level of concordance was observed across the age groups. Indeed, homeobox B13 (*HOXB13*) was the only DEG found to be significant in both tumor versus NAT analyses that was discordant for direction of effect (Supplementary Figure S1 and Table S5). 

To define the transcriptomic landscape of FAP tumors, we downloaded matched FAP tumor versus NAT data from GSE153385 and performed paired regression analyses in a manner similar to TCGA-COAD. We identified 951 of 2263 FAP tumor DEGs (42.01%) that were significant and concordant for direction of effect in our TCGA-EOCRC analysis, indicating a strong overlap for tumor related processes in the development of CRC for FAP and TCGA-EOCRC (Supplementary Table S6). 

### 3.4. Overlap between DEGs Identified in FAP Colon Organoids and EOCRC Tumors

**Figure 3:**
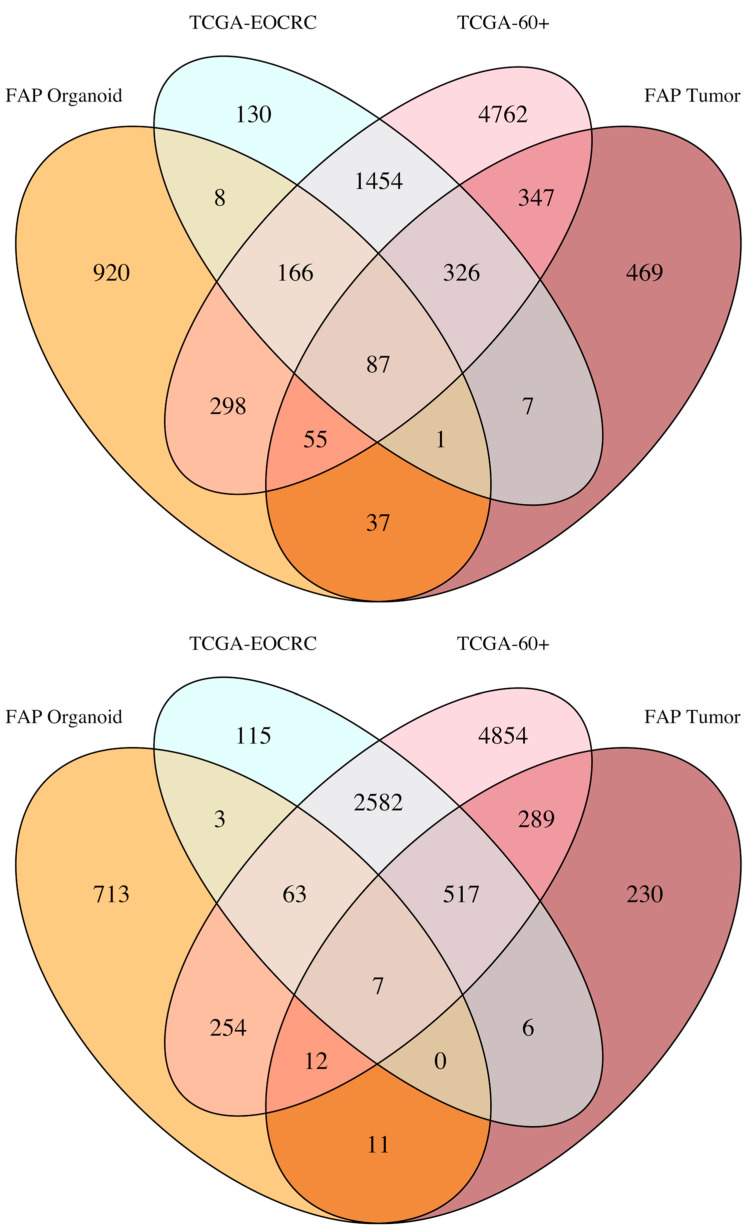
Venn diagram demonstrating the overlap of significant DEGs (FDR < 0.05) that were increased (left) and decreased (right) in FAP and CRC tumors in the four cohorts analyzed. Datasets were grouped by color: orange (FAP versus healthy colon organoids), light cyan (TCGA-EOCRC), pink (TCGA-60+) and dark red (FAP colon tumors (GSE153385)). Generalized linear models were fit for each gene in DESeq2 and a Wald test was used for the identification of significant DEGs.

To determine the extent to which our FAP colon organoid analyses modeled the gene expression differences seen in EOCRC tumors, we overlaid our novel organoid findings with both FAP and TCGA-EOCRC tumors. Of the 2635 DEGs identified in our FAP colon organoid study, 210 were differentially expressed (FDR < 0.05) in FAP tumors versus NAT (Supplementary Table S7). This represented an enrichment of FAP tumor related DEGs in our analysis of colon organoids of FAP versus healthy subjects (*p* = 1.22 × 10^−39^). Next, we found that 38.41% (1012) of FAP colon organoid DEGs were present in at least one TCGA analysis (Supplementary Table S8). Enrichment for FAP colon organoid DEGs were observed in our analyses of TCGA-EOCRC (*p* = 4.84 × 10^−8^) and TCGA-60+ (*p* = 7.17 × 10^−16^). A total of nine DEGs unique to TCGA-EOCRC were identified in our FAP colon organoid analysis. Of these nine, only *EPDR1* was also present in FAP tumors (FDR = 0.01). However, *EPDR1* (FDR = 0.063) and forkhead box O3 (*FOXO3*; FDR = 0.051) and adrenomedullin (*ADM*; FDR = 0.056) trended towards significance in TCGA-60+. Finally, by comparing data generated across all analyses, we found that 142 DEGs were significant across each analysis (Figure 3; Supplementary Table S9). The majority of these DEGs (66.20%) were concordant for direction of effect in each dataset, with *FOXQ1* being the highest cumulatively ranked DEG observed across all four analyses. 

### 3.5. Technical Validation of FAP DEGs and Confirmation of Findings in Biopsies

We performed qPCR on a subset of genes that were either among the most significant DEGs identified in our analysis of colon organoids of FAP versus healthy subjects and/or were significant in an analysis of either EOCRC tumor datasets: *NKD1, EYA2, EGR2, EPDR1* and *IGFL1*. Of the five genes chosen for technical validation, four were significant and followed the same direction of effect in our qPCR analysis (Table 1), while *IGFL1* displayed a trend towards a significant increase in FAP colon organoids (*p* = 0.052). By extending our analysis to matched FAP (*n* = 2) and healthy control (*n* = 2) colon biopsies, we found that three of the four genes (*EPDR1*, *EYA2* and *NKD1*) with detectable levels of expression were concordant for direction of effect (Supplementary Figure S2). Of note, *IGFL1* expression was not detected in FAP or healthy colon biopsies. 

### 3.6. Role of Lifestyle Factors in Driving Expression of FAP DEGs

**Figure 4:**
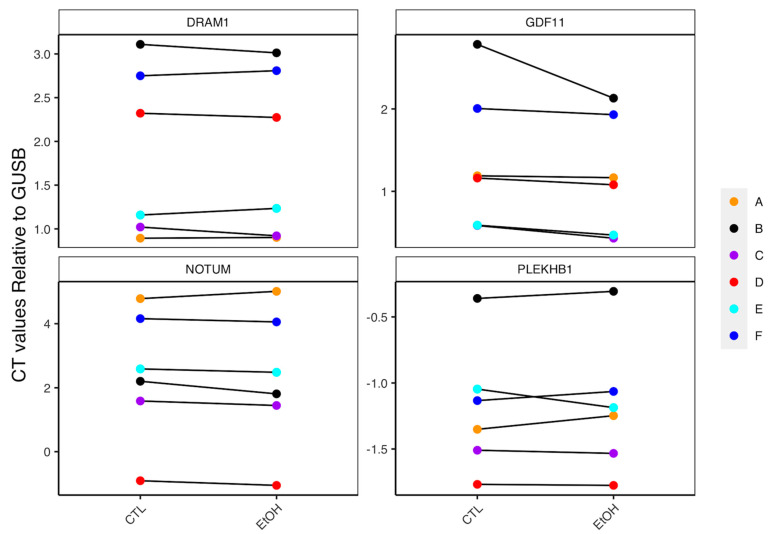
Summary of qPCR analysis from ethanol exposure of FAP colon organoid lines. CT values were generated for each gene following subtraction from internal control using a delta CT method. High CT values correspond to low expression. Reductions in CT scores between control (CTL) and ethanol treated (EtOH) FAP samples therefore represent an increase in expression for that gene in ethanol treated colon organoids. Paired samples (A–F) across treatment conditions are connected by a line to demonstrate overall direction of effect. Given the small sample size of the analysis, consistency for direction of effect was considered over significance testing to avoid the possibility that differential expression may be driven by subject-specific outliers.

A number of studies have implicated generational shifts in lifestyle factors as important contributors to the rise of EOCRC within Western populations [10]. We have previously shown that exposure of normal colon organoids to several different CRC related lifestyle factors led to widespread gene expression differences that are enriched for genes associated with CRC [18,19,20,21,22]. DEGs identified between organoids of FAP and healthy subjects were enriched for DEGs found to be differential expressed following ethanol (*p* = 1.18 × 10^−62^), aspirin (*p* = 2.07 × 10^−19^), calcium (*p* = 2.36 × 10^−32^) or carcinogen cocktail (*p* = 2.33 × 10^−77^) exposure. Importantly, several of these genes were also identified in at least one of our EOCRC analysis. High levels of alcohol consumption have previously been linked to the development of high-risk adenomas and CRC in patients with at least one existing colorectal adenoma [43]. We therefore asked if FAP colon organoids exposed to ethanol led to further changes in expression of a subset of these genes in FAP organoids. Genes selected for targeted analysis were those previously shown to be differentially expressed in ethanol exposure (*NOTUM*, *DRAM1*, *PLEKHB1* and *GDF11*) [20] and in our analysis of FAP versus healthy colon organoids. Increased expression of *NOTUM* (FDR = 9.81 × 10^−14^), *PLEKHB1* (FDR = 1.27 × 10^−3^), *DRAM1* (FDR = 3.58 × 10^−3^), *GDF11* (FDR = 0.012) were also identified in TCGA-EOCRC. Ethanol treatment led to increased expression of *NOTUM* in five of six, and increased expression of *GDF11* in all six FAP colon organoid lines (Figure 4), though effect sizes did vary. These increases were consistent for direction of effect in the larger, previous RNA-seq analysis of ethanol in healthy colon organoids [20]. More variable effects were observed for *PLEKHB1* and *DRAM1*, and larger sample sizes may be needed for the study of these genes. 

## 4. Discussion

We have used the hereditary CRC syndrome, FAP, in an attempt to provide insight into molecular mechanisms involved in EOCRC development. We observed significant enrichments for DEGs identified in our analysis of colon organoids of FAP versus healthy subjects with those seen in FAP CRC tumors versus NAT (as expected), TCGA-EOCRC and TCGA-60+, which support our use of the FAP colon organoid system to model early events preceding EOCRC development. Additional functional work will be needed to confirm whether expression of these genes drive oncogenic signaling pathways. 

We identified DEGs between colon organoids of FAP and healthy subjects that also differed consistently in an independent analysis of FAP tumor versus NAT. For example, a significant reduction in maternally expressed 3 (*MEG3*) expression was observed in both colon organoids of FAP patients and in FAP tumors. While this gene only showed a nominal decrease in TCGA-EOCRC tumors, no significant difference was observed in our analysis of TCGA-60+. Studies using CRC cancer cell lines have suggested that *MEG3* plays an important role as a tumor suppressor gene [44]. A recent study in *Apc^min^* mice of colon stem cells suggested that *MEG3* inhibits early CRC development by acting as a sponge to *miR-708* [45]. While the authors conclude that further studies modifying *MEG3* expression in CRC tumors should be considered, our data suggests that studies prioritizing the modulation of *MEG3* expression in EOCRC tumors should also be considered. 

To explore potential mechanisms underlying differential expression observed within our study, we aimed to determine the extent by which the DEGs identified here overlaid DMRs identified in our previously published, overlapping cohort of FAP and healthy colon organoids [42]. Aberrant levels of DNA methylation are considered to be a hallmark of CRC and many studies have identified widespread differential methylation in CRC tumors [16,39,46,47]. During CRC development, two seemingly contrary events unfold as extensive DNA hypomethylation and site-specific hypermethylation of DNA promoter regions lead to CRC-related events that contribute to the disease [48,49] Such differences have also led to aberrant expression of CRC-relevant genes [50] Here, we identified a subset of DEGs (*n* = 23) corresponding to previously DMRs that were also found to be specific to EOCRC [39]. This included the tumor suppressor gene, succinate dehydrogenase complex subunit D (*SDHD*), expression of which was found to be reduced in FAP colon organoids. However, while we identified differential expression in FAP colon organoids, the effects of this differential expression were greater in TCGA-60+ than TCGA-EOCRC. Despite the unique finding of differential methylation corresponding to EOCRC in previous studies [39] it is unclear whether the mechanisms driving expression of *SDHD* differ in EOCRC than later-onset CRC. Of the 23 genes that were identified, only *HLA g* was found to be at least nominally significant in TCGA-EOCRC and FAP colon organoids, but not TCGA-60+. However, given the highly diverse landscape of *HLA* and the relatively small sample sizes involved in this study, larger studies should first aim to replicate this finding. 

FAP cases are rare, but we attempt to link data generated in colon organoids of FAP patients with biopsy data generated from a small subset of matched individuals. The colon organoid model displays greatly reduced cellular heterogeneity versus the biopsies from which they were derived, given that they lack immune and stromal cell populations. This is important given the spatiotemporal nature of gene expression. Indeed, the colon organoid model is representative of the stem cell niche of the colon crypt, which is considered to be the site of CRC development [51]. Thus, performing an analysis of the specific cells implicated in CRC tumor development provides relevance and reduces the risk of confounding in downstream regression analysis that may accompany increased cellular heterogeneity. However, the time, infrastructure and specific skill set required to generate organoids from colon biopsies limit their applicability as a model for biomarker evaluation. As such, we aimed to validate five DEGs identified through our colon organoid analysis in a small subset of colon biopsies. Three of the four genes tested were concordant for direction of effect with our colon organoid analyses. This included an increase in the expression of *EPDR1* in FAP colon organoids. Increased *EPDR1* expression was most significant in EOCRC, reaching FDR correction in both FAP and TCGA-EOCRC analyses. However, this gene did trend to significance in our TCGA-60+ dataset and may therefore represent a strong target for early monitoring of CRC across age groups. That these DEGs were seen in both the colon organoid model and in a subset of colon biopsies of matched individuals highlights their relevance to CRC and the biomarker discovery potential of our study. Of interest, an increase in *IGFL1* was identified in our RNA-seq analysis of FAP colon organoids. *IGFL1* has previously been proposed to be an oncogene in other cancers, where it was found to aid in cancer cell growth and survival [52]. This increase was not found within TCGA-60+, but was identified in our TCGA-EOCRC analysis. Importantly, qPCR analysis of FAP colon biopsies was unable to detect expression of *IGFL1*. However, we were able to provide a partial technical validation for this finding in qPCR analysis of colon organoids of FAP versus healthy subjects. Tumor samples are known to have increased stemness scores versus matched biopsies [53], whereas colon organoids represent an important model of the colon stem cell niche. It is therefore possible that *IGFL1* plays an important role in stem cells and/or other undifferentiated cell populations that are generally of low abundance in normal biopsies. Indeed, previous studies have found that *IGFL1* expression is primarily limited within normal tissues that contain a high abundance of undifferentiated cells, such as fetal tissue and the spinal cord [54]. Importantly, IGFL sequences are not well conserved between mice and human [55], limiting its potential for modelling in other species. The identification of increased *IGFL1* expression in FAP colon organoids therefore supports the use of the organoid model for studying EOCRC. Future research would benefit from the availability of larger, publicly available EOCRC datasets for comparison. These datasets could be used to help better define DEGs unique to EOCRC development. 

Previously, we have shown that exposure of CRC lifestyle factors leads to modified gene expression of CRC-related genes [18,19,20,21,22]. Given the strong enrichment of the FAP DEGs observed here and CRC, we were not surprised to see that FAP DEGs were also enriched for targets of various CRC lifestyle factors. Importantly, one theory attempting to explain the rise in EOCRC rates observed across recent generations centers on changes in Western lifestyle, including increased alcohol consumption [10]. As such, we performed qPCR analysis on a subset of genes previously found to be altered following ethanol treatment that were also differentially expressed in FAP colon organoids and in at least one EOCRC dataset. We identified a relatively consistent increase in the expression of *NOTUM*, as well as a consistent increase in the expression of *GDF11*. These findings were in line with the direction of effect observed in a larger analysis of normal colon organoids and represent a subset of EOCRC relevant DEGs whose expression are further perturbed following exposure to at least one common Western lifestyle factor. *GDF11* has also previously been associated with aging in numerous studies; however, its exact pathophysiological role remains unclear [56]. Increased *GDF11* has been associated with increased stemness [57], and CRC patients with increased expression of this genes have been shown to have reduced survival [58]. Silencing *GDF11* in the CRC cell line Caco-2 has also implicated a role for this gene in proliferation [57,59]. These findings add further weight to the potential role of known lifestyle factors in EOCRC. Further lifestyle factor exposure studies in organoids may yield improved insight into their role in both FAP EOCRC tumor development. 

There are several limitations to our study. The first is the sample size of the datasets considered here. FAP sample size considerations were largely limited by the rarity of this syndrome in the population. Future studies should aim to build upon these findings through the use of larger cohorts. Second, we identify established modifiable targets of CRC relevant lifestyle factors through a limited selection process of genes that were also found to be related to CRC. It is unknown whether long term culture under these exposures may affect these genes further, or whether other genes that are relevant to CRC may be better identified. Further research is needed to explore how these genes may contribute to CRC risk. Third, we identify numerous genes relevant to FAP and TCGA-EOCRC tumorigenesis that are also seen in colon organoids of FAP patients in the absence of cancer. However, it remains to be determined whether these DEGs reflect useful early biomarkers for EOCRC tumorigenesis and whether they have actionable potential as future drug targets. Further research will be needed in relevant models of CRC to determine whether these genes actively contribute to tumorigenesis. Determining whether these transcriptomic events are also present in blood would constitute an approach that would be more readily accessible and less invasive than biopsies collected at colonoscopy. Fourth, the normal-appearing FAP mucosal biopsies from which colon organoids were derived may have contained some, undetected microadenomas. Finally, inconsistent evidence for the role of APC in non-hereditary EOCRC exists. For example, the rate of APC mutations at known hotspot regions has been shown to be greater in non-hereditary, non-EOCRC populations [60]. However, sequencing of the *APC* gene in a small cohort of EOCRC patients found that mutation rates across the gene occurred at a similar level as proposed in non-hereditary, non-EOCRC populations. Most importantly, the authors conclude that the pattern of these mutations were more similar to inherited *APC* mutations than to non-hereditary, later onset CRC [60].

In conclusion, we have identified widespread differential expression in colon organoids of FAP and healthy subjects. These DEGs were enriched for genes related to EOCRC. Our findings highlight potential early markers for EOCRC, but also mechanisms through which lifestyle factors may further modulate the expression of genes implicated in EOCRC.

## Figures and Tables

**Table 1:** Summary of qPCR findings for selected genes chosen for technical validation. CT values were generated for each gene following subtraction from internal control. High CT values correspond to low expression. Negative effect size estimates and test statistics therefore indicate increased expression of a gene in FAP versus healthy colon organoids.

Gene	Estimate	Standard Error	Test Statistic	*p*
*NKD1*	−6.069	1.012	−6.001	5.42 × 10^−4^
*EGR2*	−3.139	0.653	−4.805	1.95 × 10^−3^
*EYA2*	3.820	1.326	2.880	0.024
*EPDR1*	−2.054	0.823	−2.496	0.047
*IGFL1*	−2.697	1.116	−2.417	0.052

## Data Availability

All data has been made available to Gene Expression Omnibus and can be accessed through accession number: GSE207398.

## Supplementary Materials

The following supporting information can be downloaded at: https://www.mdpi.com/article/10.3390/cancers14174138/s1, Supplementary Table S1: Relevant demographic information of the colon organoids used within this study. Supplementary Table S2: RNA-seq mapping statistics for all novel samples used in the study. Supplementary Table S3: Pathway enrichment analysis of significant DEGs identified in regression of FAP versus healthy colon organoids. Supplementary Table S4: Summary of RNA-seq findings for a subset of genes for which DMRs had previously been identified. Positive fold changes represent genes that were increased in FAP versus healthy colon organoids. Supplementary Table S5: Summary of RNA-seq findings for analysis of non-hereditary EOCRC and non-EOCRC regression models. Positive fold changes represent genes that were increased in tumor versus NAT. Cumulative rank was generated by first ranking significant DEGs in each analysis in order of greatest significance and summing the total of these two values. A rank was then ordered based on the subsequent lowest scores. Supplementary Table S6: Summary of consistent RNA-seq findings identified in FAP tumor and non-hereditary EOCRC regression models. Positive fold changes represent genes that were increased in tumor versus NAT. Cumulative rank was generated by first ranking significant DEGs in each analysis in order of greatest significance and summing the total of these two values. A rank was then ordered based on the subsequent lowest scores. Only DEGs that were significant in both analyses were reported. Supplementary Table S7: Summary of RNA-seq consistent findings identified in FAP colon organoid and FAP colon tumor regression models. Positive fold changes represent genes that were increased in FAP versus healthy colon organoids and/or tumor versus NAT. Cumulative rank was generated by first ranking significant DEGs in each analysis in order of greatest significance and summing the total of these two values. A rank was then ordered based on the subsequent lowest scores. Only DEGs that were significant in both analyses were reported. Supplementary Table S8: Summary of RNA-seq findings identified in FAP colon organoids and both non-hereditary EOCRC regression models. Positive fold changes represent genes that were increased in FAP versus healthy colon organoids and/or tumor versus NAT. Cumulative rank was generated by first ranking significant DEGs in each analysis in order of greatest significance and summing the total of these three values. A rank was then ordered based on the subsequent lowest scores. Supplementary Table S9: Summary of significant DEGs identified from RNA-seq in each analysis. Positive fold changes represent genes that were increased in FAP versus healthy colon organoids and/or tumor versus NAT. Cumulative rank was generated by first ranking significant DEGs in each analysis in order of greatest significance and summing the total of these three values. A rank was then ordered based on the subsequent lowest scores. Only genes that were concordant for direction of effect in each analysis were ranked. Supplementary Figure S1: Volcano plot of DEGs identified in FAP versus healthy colon organoid analysis (*n* = 13) that demonstrates highlights similarity of differential expression reporting in EOCRC tumor datasets. Genes were separated into five categories. Black genes (N.S) were those that did not reach any significance level. Orange genes reached FDR significance, but were unique to FAP colon organoids. Dark red genes were significant in colon organoids and TCGA-EOCRC. Purple genes were significant in colon organoids and FAP tumors. Blue genes were significant in colon organoids, TCGA-EOCRC and FAP tumors. Generalized linear models were fit for each gene in DESeq2 and a Wald test was used for the identification of significant DEGs. Positive log2 fold changes represent genes that were increased in FAP versus healthy colon organoids. Supplementary Figure S2: QPCR analysis of FAP (*n* = 2) versus healthy colon biopsies (*n* = 2). Following normalization to internal control, the relative expression of each gene, for each FAP patient was calculated against the average expression of both healthy controls. Values greater than one indicated increased expression in FAP colon biopsies. *IGFL1* expression was not detected in any biopsy.
