# Supplementary figures and images for: Insights into Early Onset Colorectal Cancer through Analysis of Normal Colon Organoids of Familial Adenomatous Polyposis Patients
Source: Cancers (Basel). 2022 Aug 26;14(17):4138. doi: 10.3390/cancers14174138 (PMC9454756; doi:10.3390/cancers14174138)

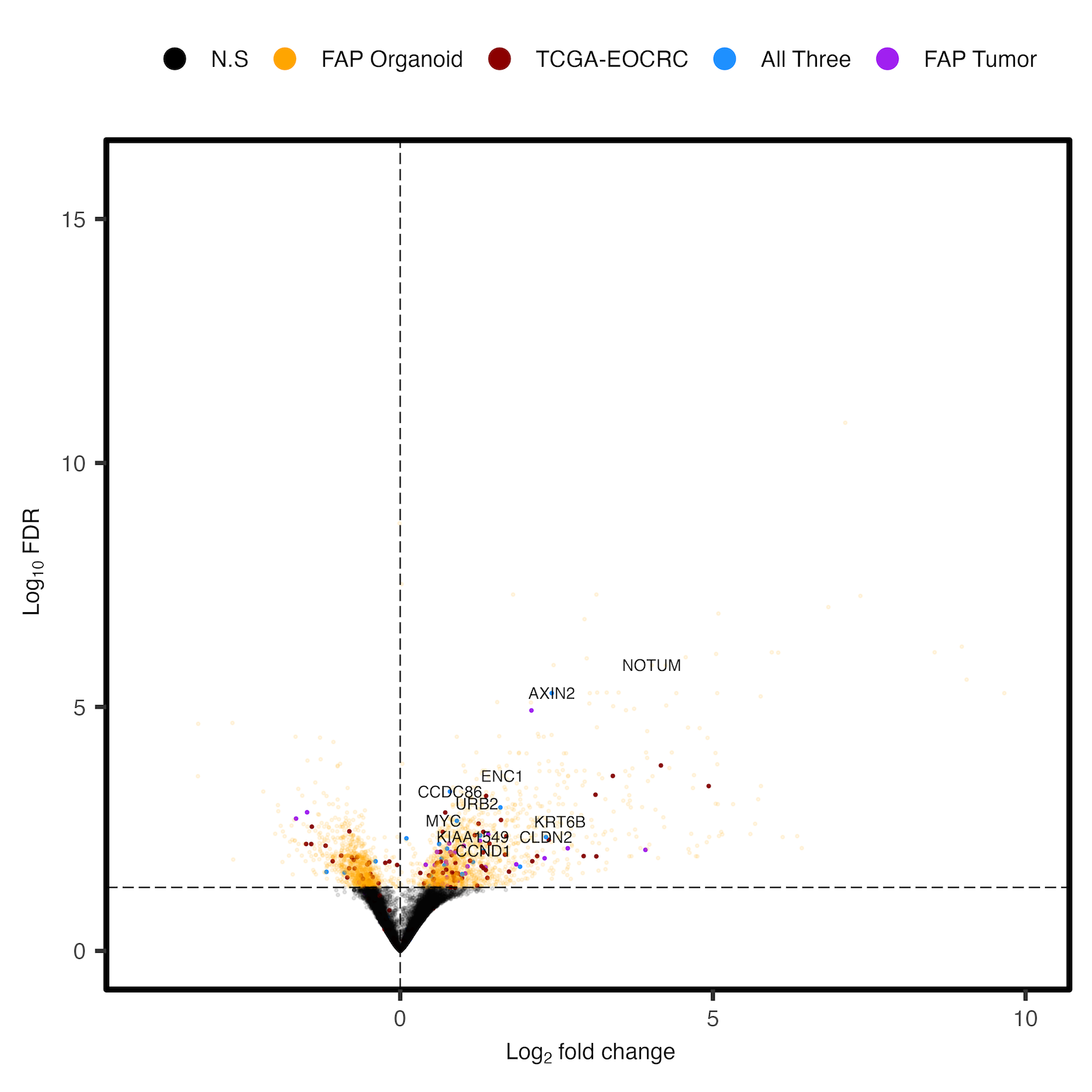

Supplement: Supplementary file 1 [file cancers-14-04138-s001.zip › FigureS1.tiff]

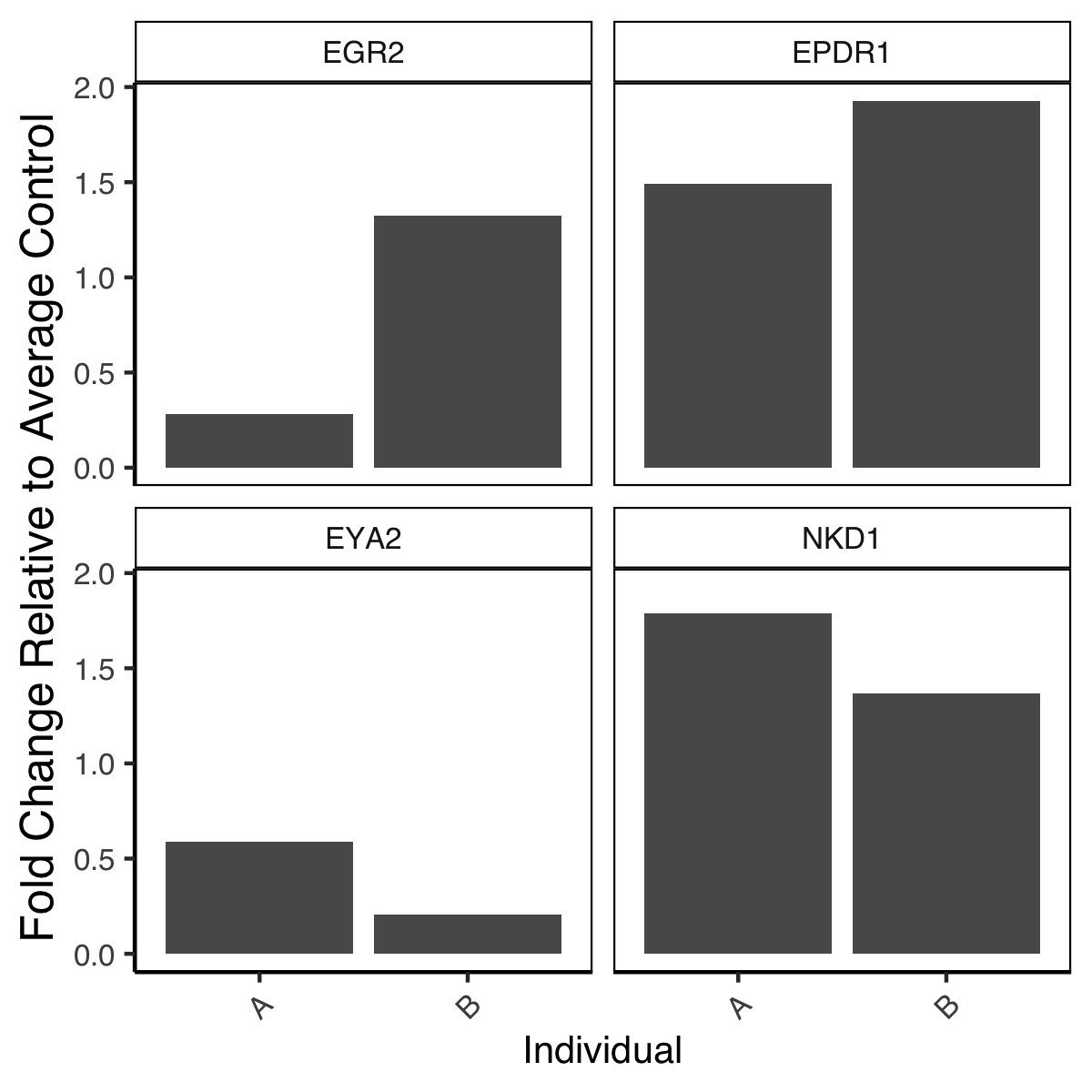

Supplement: Supplementary file 1 [file cancers-14-04138-s001.zip › FigureS2.tiff]
